# Tumor Metastasis to the Oral Soft Tissues and Jaw Bones: A Retrospective Study and Review of the Literature

**DOI:** 10.1002/cre2.70011

**Published:** 2024-10-17

**Authors:** Atessa Pakfetrat, Zohreh Dalirsani, Nasrollah Saghravanian, Kazem Anvari, Sajede Asalian, Armaghan Salehi, Mahboobeh Taherizadeh

**Affiliations:** ^1^ Oral and Maxillofacial Diseases Research Center Mashhad University of Medical Sciences Mashhad Iran; ^2^ Department of Radiotherapy Oncology and Cancer Research Center Mashhad University of Medical Sciences Mashhad Iran; ^3^ Mashhad University of Medical Sciences Mashhad Iran; ^4^ Student Research Committee, Faculty of Dentistry Mashhad University of Medical Sciences Mashhad Iran; ^5^ School of Health Mashhad University of Medical Sciences Mashhad Iran

**Keywords:** jaw metastasis, oral cancer, oral metastasis

## Abstract

**Objectives:**

Metastasis to the oral soft tissues and jaw is rare and accounts for 1%–3% of maxillofacial malignancies. These lesions usually occur in the context of an extensive malignant tumor with a poor prognosis.

**Materials and Methods:**

Archived cases from the Oral and Maxillofacial Pathology Department of the Faculty of Dentistry and two hospital centers of Mashhad University of Medical Sciences were examined. Inclusion criteria were cases with available records of pathologically confirmed metastatic lesions of the oral cavity with or without diagnosed primary malignancy.

**Results:**

Metastatic lesions in the oral cavity and jaw were found in 18 patients, including seven women and 11 men, with a mean age of 49.5 years. Metastatic lesions were more common in the jaw (66%) and particularly in the mandible (38%) than elsewhere. In the case of soft tissue metastases, the gingiva was more affected than other sites. The primary tumor was most commonly in the kidney in men and in the breast in women (36%–28%). In addition, the diagnosis of a metastatic lesion led to the detection of the primary tumor elsewhere in six out of 18 cases (33.3%).

**Conclusions:**

Early diagnosis of the lesions is challenging, given the absence of specific signs or symptoms, which, in some cases, nonetheless resemble inflammatory, benign, reactive lesions. Therefore, dentists play a crucial role in diagnosing such lesions, as they lead to the discovery of hidden distant primary tumors. Biopsy should always be considered for suspicious lesions, even if the probability is very low.

## 1 Introduction

Malignant lesions of the oral cavity account for 5%–6% of human cancers (Bertoin and Baudet‐Pommel 1997; Zachariades 1989). Metastasis to the oral cavity is rare due to various filtration systems that prevent the spread of metastasis (Batson 1940; Hirshberg et al. 2014)

Metastatic neoplasms constitute 1%–3% of maxillofacial malignancies, usually indicate disseminated disease with a poor prognosis of several months, and have a worse prognosis compared to metastases in other parts of the body (Lim et al. 2006; Kirschnick et al. 2022; Lee and Lee 2017; Kumar and Manjunatha 2013). Therefore, early cancer diagnosis is of great importance and improves survival (de Carvalho Kimura et al. 2022). The average time between the discovery of the primary tumor site and metastatic invasion is 40 months (Hirshberg et al. 2008).

Most metastatic lesions of the oral cavity occur between the ages of 40 and 70 years (Lim et al. 2006; Lee and Lee 2017). Studies show that metastases in the oral cavity and jaw occur more frequently in men (Lim et al. 2006; de Carvalho Kimura et al. 2022). Metastases to the jaw, particularly the mandible and its posterior, are more common than metastases to the oral soft tissues. In the case of metastases to the oral soft tissues, the gingiva is the most common location (Hirshberg et al. 2008).

In a 2020 systematic review, the most common primary sites of tumors metastasized to the jaw or oral soft tissues were lung, breast, kidney, skin, and liver (Kirschnick et al. 2022). The type of primary tumor and the area that metastasizes to the oral cavity are different among men and women. The most common primary tumors are lung, kidney, liver, and prostate cancer in men and breast, genitalia, kidney, and colorectal cancer in women (Hirshberg et al. 2008; Seoane et al. 2009; Watters et al. 2021; Glick 2015). In most cases, maxillofacial metastases lead to death (Zachariades 1989; Clausen and Poulsen 1963; Hirshberg, Leibovich, and Buchner 1994; McClure et al. 2013).

Because oral metastases are rare, diagnosing them and localizing their origin are difficult for both physicians and pathologists (Hirshberg et al. 2008). The diagnosis of these lesions, especially soft tissue metastases, can be challenging, as their clinical manifestation sometimes mimics reactive lesions such as hyperplasic swellings, including pyogenic granuloma (PG), *epulis fissuretum*, *etc*. (Shen et al. 2009; Nuyen and Tang 2016; Maschino et al. 2013; Vasilyeva et al. 2018). In addition, routine skeletal examinations to assess metastasis may not detect metastatic jaw tumors, as these examinations rarely involve the jaw, and metastatic lesions in the oral cavity, on the other hand, show no specific signs or symptoms (Nawale et al. 2016).

The diagnosis of these lesions is of paramount importance, especially when it leads to the detection of primary malignancies in other parts of the body. Sometimes, the first signs of metastasis in the oral cavity are detected when the primary tumor has not yet been identified (van der Waal, Buter, and van der Waal 2003).

Despite numerous epidemiological studies on oral metastases worldwide, studies on the Iranian population are very limited. To the best of our knowledge, only one retrospective study of head and neck metastases has been conducted in the Iranian population, of which only four involved oral and jaw metastases (Sadri et al. 2015). The aim of this study was to investigate metastatic lesions in the oral cavity, their symptoms and clinical manifestations, and demographic data from diagnostic and therapeutic centers in Mashhad, Khorasan Razavi Province, Iran.

## 2 Subjects and Methods

This retrospective cross‐sectional study was conducted from 2020 to 2022 on the records of patients diagnosed with metastatic lesions in the oral cavity and jaw from 1970 to 2021, available at the Department of Oral and Maxillofacial Pathology, Faculty of Dentistry, as well as two main cancer hospital centers in Mashhad University of Medical Sciences, Iran.

The inclusion criteria were patients with (1) records of clinical and, if necessary, radiographic characteristics, metastatic lesions of the oral cavity with a history of malignancy and histopathological similarity to the primary tumor or (2) microscopic findings confirming the presence of a metastatic lesion without identifying the site of the primary malignancy. Patients with lesions due to direct invasion of the primary tumor were excluded from the study. In addition, patients with metastases in the major salivary glands, lymph nodes, and facial skin, as well as cases with insufficient information were excluded from the study.

First, the pathology reports were checked and, if necessary, the pathologist was consulted. Next, the metastatic cases of 968 patients with malignant tumors of the oral soft tissues and jaw were extracted. Cases in the first 5 years of the period 2012–2021 were extracted via keyword search from the archives of the Pathology Department of Qaem General Hospital. As the keyword search in the database was limited to this period only, ICD criteria were used to search for metastases over the next 5 years.^1^

ICD criteria were also used for the records of Omid Oncology Hospital Center in Mashhad during the same period. In this center, the archive of outpatients was also searched for diagnostic cases with metastases in the oral cavity and jaw. Next, a checklist of clinical features was created, including age at diagnosis, gender, primary malignancy and metastatic tumor locations, and primary malignancy and metastatic tumor treatments. In addition, the clinical profile of oral cavity metastases as well as the histopathological diagnosis and the time between the diagnosis of the primary tumor and metastatic lesion were recorded.

The data were analyzed using SPSS 20.0 and descriptive statistics, including central indicators, dispersion, and frequency distribution, were used.

## 3 Results

**Figure 1:**
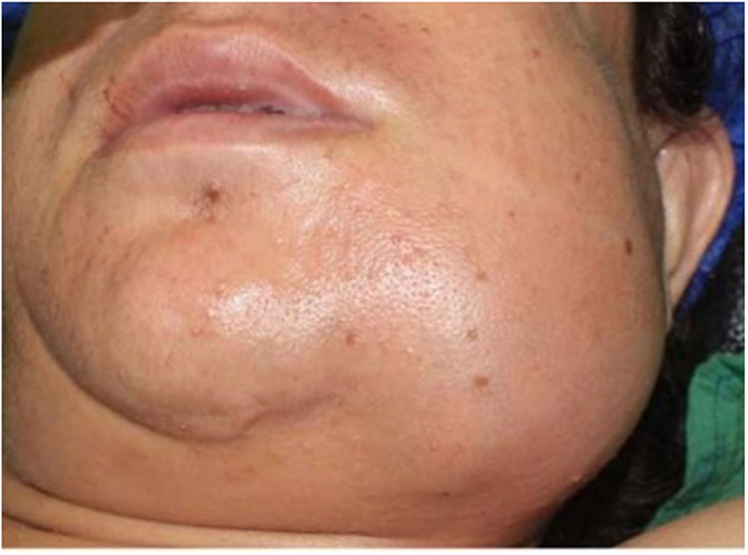
Metastatic pheochromocytoma in the mandible of a middle‐aged woman who had previous pheochromocytoma in her adrenal gland.

**Figure 2:**
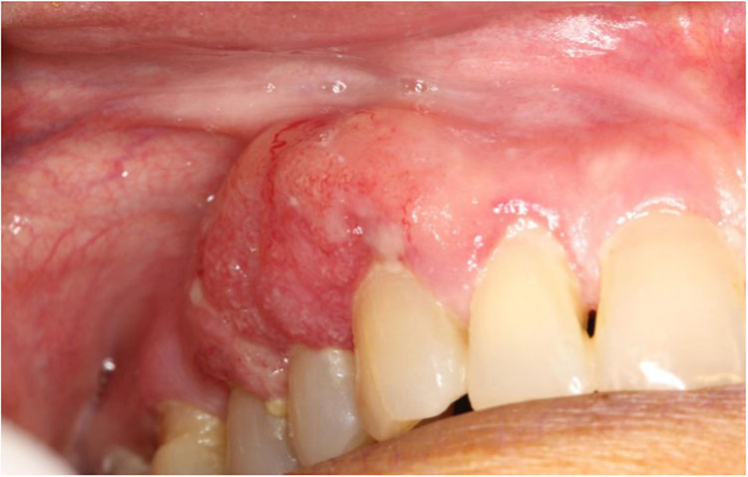
Metastatic adenocarcinoma in a woman with a history of colon adenocarcinoma.

**Figure 3:**
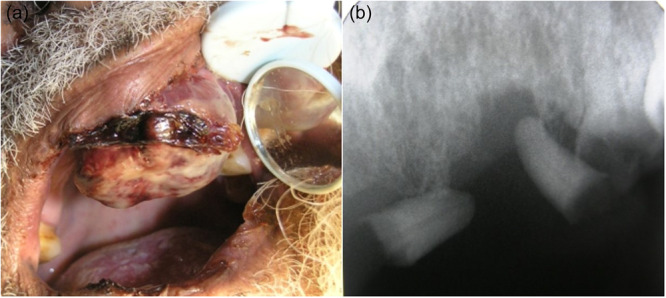
Metastatic renal cell carcinoma in the maxilla of a 75‐year‐old man who had previous renal cell carcinoma. (a) Clinical view and (b) bone resorption in X‐ray.

**Figure 4:**
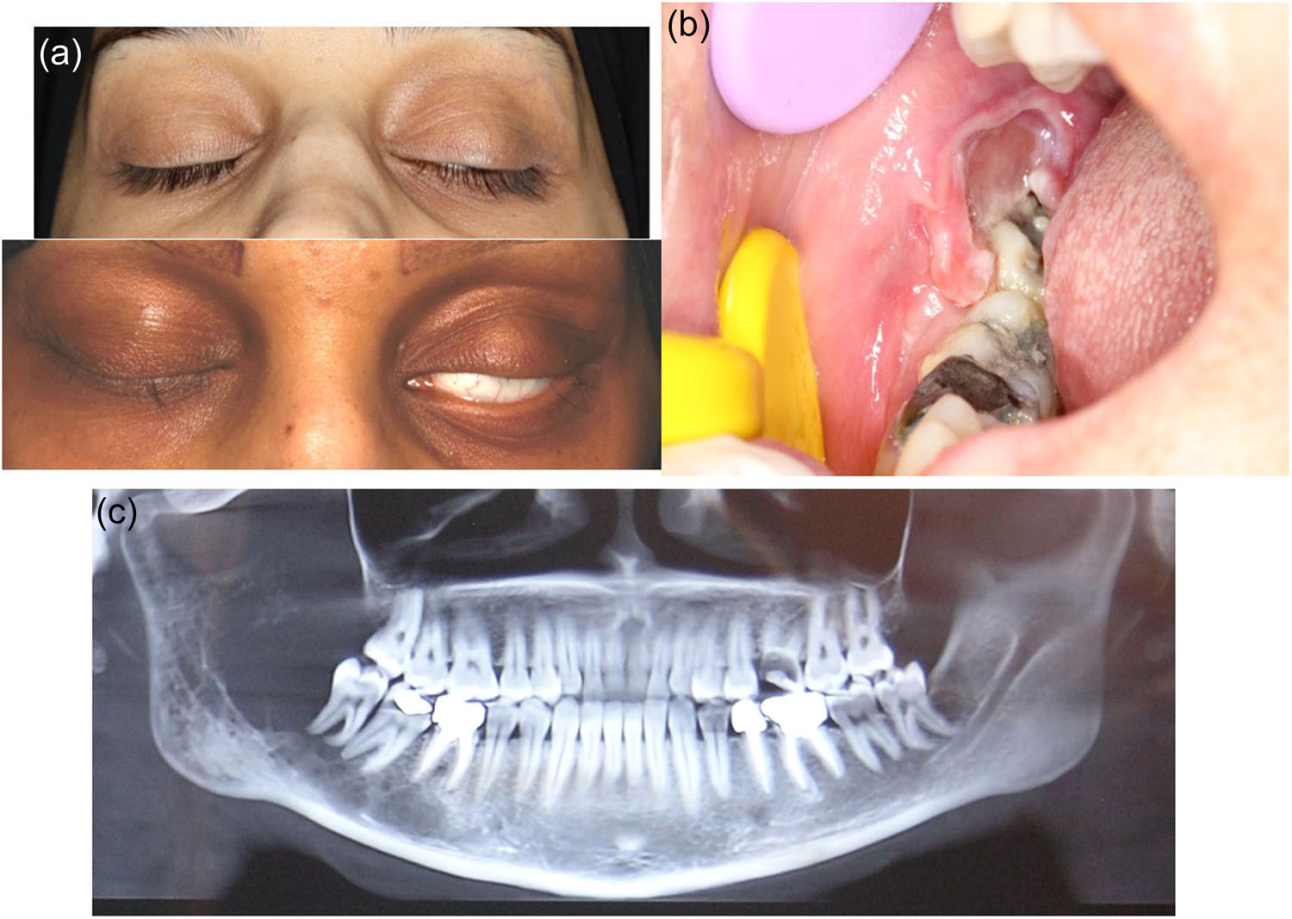
Metastatic carcinoma in a young woman without a history of cancer. She had swelling of the right cheek and toothache (right lower third molar) for a month and right chin's skin paresthesia for eight months before her first visit. She lost about 30 kg of weight within 10 months. She was also suffering from chest, leg, and back pain and (a) inability to close the left eyelid. Antibiotic and NSAIDs had been prescribed by a dentist and a physician before she was referred to a dental school. Breast cancer was discovered after necessary analyses as the primary cancer and widespread metastases were found. (b) Intraoral view. (c) Presence of radiolucency in the involved area in the panoramic radiograph.

**Figure 5:**
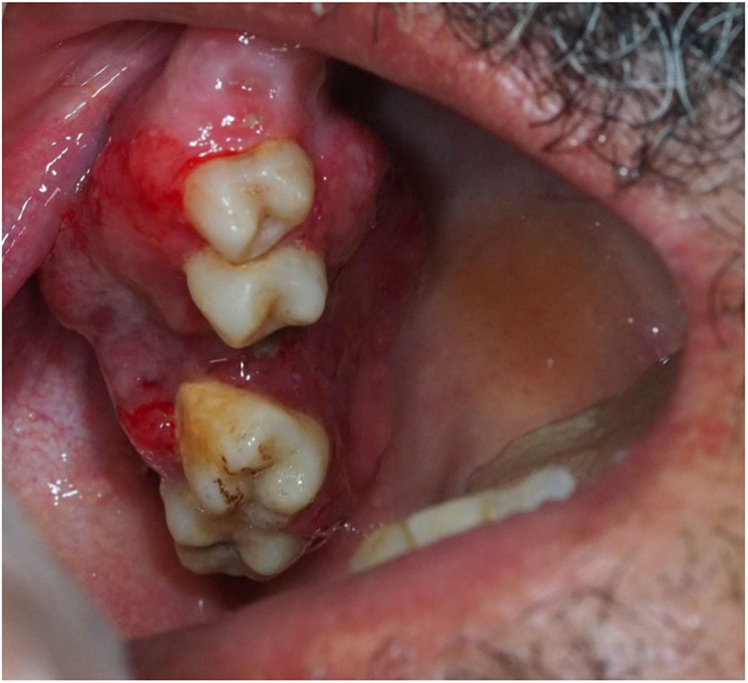
Metastatic adenocarcinoma in a patient with colon cancer.

**Figure 6:**
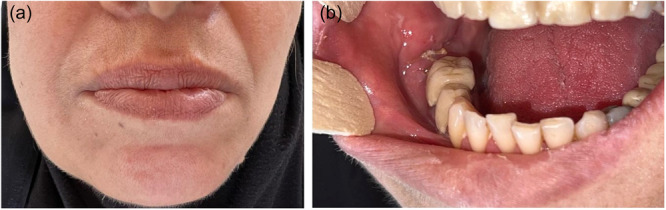
Metastatic adenocarcinoma in the posterior mandibular region in a patient who had no history of cancer. After full‐body paraclinical analysis, lung cancer was discovered as the primary tumor. (a) Extraoral view and (b) intraoral view.

Examination of the records from a period of 52 years in one dental center and from a period of 10 years in two hospital centers identified 18 patients with histopathologically confirmed oral and maxillofacial metastases with the origin of a primary tumor at a remote site. Among the 968 cases of malignancy patients examined, 13 had a metastatic tumor originating from the primary malignancy at a remote site, accounting for about 1.3% of all oral and maxillofacial malignancies at the center. Demographic information and details of the clinical features of 18 patients are presented in Table S1, and some of cases are shown in Figures 1, 2, 3, 4, 5, 6.

### 3.1 Demographic Information

Oral metastases were more common in men (11/18, 61.1%) than in women (7/18, 38.9%) (1.6:1). The mean age of the patients was 49.5 years (range 6–83). The patients were predominantly between 40 and 60 years of age. The mean age of patients with jaw metastases (46.21) was lower than that of patients with soft tissue metastases (61.25). In addition, the mean age in men and women was almost the same (48.9 and 50.6 years). The mean age of patients with metastases in the mandible (40.25 years) was lower than that of patients with metastases in the maxilla (56.25 years). In the case of jaw metastases, there were twice as many in men as in women, whereas in the case of oral soft tissue metastases, the number was the same.

### 3.2 Clinical Features and Symptoms

Metastatic lesions were more common in the jaw (12 cases, 66%), particularly the mandible (eight cases, 44.4%), compared to other sites. In addition, four cases of metastases (22.2%) were peripheral and two cases (11.1%) were peripheral with underlying bone involvement. Among the soft tissue metastasis, the gingiva and alveolar mucosa were more affected than other sites.

In most patients, the predominant clinical sign was the appearance of a mass and the main symptom was pain (40%–50%), followed by neurological symptoms such as paresthesia (30%) and, in some cases, symptoms such as bleeding, luxation, and displacement of teeth (28.6%).

### 3.3 Radiographic Features

Among the central lesions, the radiographic feature was radiolucency in six cases and osteogenic radiolucency in one case. Information on the radiographic features of other cases of central/peripheral lesions was not available.

### 3.4 Histopathological Features

In histopathological view, the presence of neoplastic cells with changes like hyperchromatism, polymorphism, increased mitotic activity with atypical mitoses, increased nuclear/cytoplasmic ratio, and cellular fields such as the presence of clear cells lead to diagnosis of malignancy (like renal cell carcinoma) with the probability of the presence of metastatic lesions, especially in patients with a previous history of malignancy who had a new lesion in the oral cavity.

In order to identify the similarity between the oral lesion's cells and the primary tumor's cells, immunohistochemical staining was performed. To diagnose the epithelial origin of the lesions and distinguish between different types of metastatic carcinomas, types of CYTOKERATIN specially 20 and seven was used and VIMENTIN was also used to diagnose the mesenchymal origin of the lesions. Also, MELAN A and HMB45 were used for the lesions suspected to be melanoma and S100 staining for suspected neurological lesions. Histochemical staining such as PAS and MUCICARMIN was also used for more accurate diagnosis. Table 1 shows the histomorphologic information and IHC panels that led to the diagnosis of each case.

**Table 1:** Histomorphologic information and IHC panels of cases.

Histopathologic diagnosis of metastatic lesions	Primary site	Histomorphologic information	Immunohistochemical panels
Adenocarcinoma	Lung	Acinar type/gland formation/increased fibrous stroma.	PDL1 (POS)
Adenocarcinoma	Colon	Moderately differentiated gland formation with a cribriform pattern.	CK20 (POS) CK7 (NEG)
Adenocarcinoma	Stomach	Tubular type with nuclear abnormalities/high N/C/polymorphism/increased mitotic figure.	CK20, CK7 (POS) ESTROGEN RECEPTOR, PROGESTRON RECEPTOR (NEG)
Adenocarcinoma	Prostate	Glandular crowding with an infiltrative invading pattern with an intraluminal crystalloid body and nuclear atypia.	PSA (high POS) NKX3 (POS) CK7, CK20, TTF1 (NEG)
Round cell carcinoma/clear cell carcinoma/renal cell carcinoma	Kidney	Tumor cell with abundant vacuolated cytoplasm with indistinct borders and nuclear atypia of prominent nucleoli.	PAX8, PAX2, VIMENTIN, EMA (POS) CK20/MELAN A (NEG)
Squamous cell carcinoma	Esophagus	Invasion of malignant sheets of squamous cells with nuclear hyperchromatism and polymorphism with keratin pearls and individual cell keratinization fibrous stroma and infiltrative response.	NO NEED
Osteosarcoma/small round cell tumor	Leg	Tumor cell with densely eosinophilic cytoplasm larger than osteoblast with nuclear atypia/osteoid‐like pattern/thin and lace‐like and irregular trabecula.	S100 OSTEOCALCIN, OSTEOPONTIN (POS)
Follicular carcinoma/thyroid carcinoma	Thyroid	Trabecular pattern of small‐sized follicles with no necrosis that mimic thyroid tissue/nuclear atypia and mitotic figures (less than three/10 in a high‐power field).	TTF1 (POS)
Neuroblastoma/round cell tumor	kidney	Small round blue cell tumor with a small amount of cytoplasm and dark dens chromatin with coagulation necrosis.	NSE, CD57, CD 56, (POS) EMA, HMB45, DESMIN (NEG)
Ductal carcinoma	Breast	Papillary frons containing prominent fibrovascular septa projection/intraductal lumen with lack of myoepithelial cells.	CK 5/6/P40/P63 (POS) For myoepithelial cells. E CADHERIN ESTROGEN and PROGESTRON receptor (POS). Also CK5, CK6, CK14 (NEG) for acinar cells.
Thymic carcinoma	Thymus	Nest of large polyhedral cells with intercellular bridges/vesicular and hyperchromatic nucleoli in keratinizing form.	KERATIN, CD57, CD70, EMA (POS) VIMENTIN (NEG)
Pheochromocytoma	Adrenal gland	Nested (Zellballen) arrangement with large polygonal extensively vacuolated cells and abundant granular cytoplasm and uniform nucleoli.	Chromogranin, SYNAPTOPHYSIN, S100, CD10 (POS) CALRETININ, MELAN A, HMB45 (NEG)

Abbreviations: NEG, negative; POS, positive.

### 3.5 Primary Tumor's Site

The most common site of the primary tumor was the kidneys (4/18, 22.2%), followed by lung, breast, esophagus, and colon with equal prevalence (2/18, 11.1% each). Other primary tumors were in the adrenal gland, thymus, thyroid, stomach, leg and prostate (one case each, 5.5%). Interestingly, all renal tumors metastasized to the oral cavity were in men (4/11, 36.4%). In women, the breast was the most common site of primary tumor, with a frequency of 28.5%.

Kidney and breast tumors had metastasized to the jaw, particularly the mandible (three out of four cases and two out of two cases). In addition, three out of four cases of metastases to the oral soft tissue were due to cancers of the gastrointestinal tract, that is, esophagus, colon, and stomach. One‐third of the cases had metastases in organs other than the oral cavity, before or after the diagnosis of oral metastases.

### 3.6 Other Results

The mean time interval between the diagnosis of the primary and metastatic tumors was 3.2 years, ranging from zero to 13 years. In five of 18 cases (27.7%), the oral metastatic lesion was diagnosed before the diagnosis of the primary tumor (metastatic lesion was diagnosed by a biopsy study). In other cases, however, the primary tumor was already diagnosed and the patient was receiving treatments.

The most common histological diagnosis was adenocarcinoma, with a frequency of 33.3% (6/18), followed by renal cell carcinoma, with a frequency of 16.7% (3/18). In addition, despite the rarity of sarcoma in metastatic lesions of the oral cavity, there was one case of osteosarcoma in this study.

### 3.7 Information on Treatment and Prognosis

Most patients with metastatic lesions had received chemotherapy, radiotherapy, or a combination of both (6/16, 37.5%). In addition, 31.2% (5/16) underwent surgery with adjuvant treatments and 25% (4/16) received surgical treatment only. One patient had also received symptomatic treatment and, for two patients, information on treatment after diagnosis was not available.

The records of patients who had been followed up after diagnosis were reviewed for outcome. Of the 18 total cases, only nine patients were followed up for a year or less, six of them had died, two had recovered, and one was still under treatment.

## 4 Discussion

Metastasis to the oral cavity is very rare and has been reported in various studies to be 1%–1.5% of malignant neoplasms of the oral cavity (Hirshberg et al. 2014; Kumar and Manjunatha 2013; Kaplan et al. 2019; Shimono et al. 2021). Thiele et al. reported that 2.39% of oral and cranio‐maxillofacial malignancies in the German population were metastatic lesions. This was 1%–3% in Meyer and Shklar (1965); Thiele et al. (2011) Similarly, in our study, metastatic lesions accounted for 1.3% of all oral cavity and maxillofacial malignancies.

In this study, most of the patients were between 40 and 60 years of age. Previous studies have reported that most patients with metastatic lesions in the oral cavity are in their fifth to seventh decades of life (Hirshberg, Leibovich, and Buchner 1994; Hirshberg and Buchner 1995; Nishimura et al. 1982; Schwartz, Mignogna, and Baredes 1988). The mean age of patients with jaw metastases was lower than that of patients with soft tissue metastases in the oral cavity (46 vs. 61 years), consistent with recent studies (Lee and Lee 2017; Hirshberg et al. 2008). Van der Waal et al. reported a lower mean age in women compared to men in the Netherlands (53 and 66 years, respectively) (van der Waal, Buter, and van der Waal 2003). In our study, the mean age of both sexes was almost the same, but slightly higher in women (50.6 compared to 48.9), which cannot be evaluated statistically due to the small number of cases.

There is still disagreement about gender preference in oral metastasis. In our study, the lesions were more common in men (1.6:1), consistent with most studies (Lim et al. 2006; Lee and Lee 2017; Murillo et al. 2013). However, Nishimura et al. reported a ratio of 1:1.51, with a predominance of women in Japan in their literature review in 1982 (Nishimura et al. 1982). In our study, jaw metastases were twice as common in men as in women, which was inconsistent with other studies, including Schwartz et al. in 1988 in the US population, who reported a higher prevalence in women, as well as Kumar et al. in 2013 in India, who reported an equal prevalence (Kumar and Manjunatha 2013; Schwartz, Mignogna, and Baredes 1988).

The higher prevalence of metastases in women in the study of Schwartz et al. was due to the predominance of lesions with primary breast tumors (Schwartz, Mignogna, and Baredes 1988). In our study, the prevalence of metastases to the oral soft tissue was equal in men and women. However, Lee et al. and Kumar et al. reported a higher prevalence in women in Korea and men in India, respectively (Lee and Lee 2017; Kumar and Manjunatha 2013). Maschino et al. found a higher prevalence of metastasis to the oral mucosa in men than women in France, with or without jaw involvement. One reason for such a higher prevalence could be that lung cancer is the predominant primary tumor in patients (Maschino et al. 2013). In addition, different patterns of referral to treatment centers around the world as well as the nature of primary tumors may have an impact on gender difference in metastatic tumors.

Most studies have confirmed that metastases to the jaw are more common than those to the soft tissue of the oral cavity (Lim et al. 2006; Lee and Lee 2017; Hirshberg et al. 2008; Seoane et al. 2009; McClure et al. 2013; Shen et al. 2009; Maschino et al. 2013; Kaplan et al. 2019; Shimono et al. 2021; Murillo et al. 2013; D'Silva et al. 2006; Jham et al. 2011; Bodner et al. 2006; Owosho et al. 2016; Aniceto et al. 1990; Ho et al. 2022; Barnes 2009). Metastases to the mandible, especially the premolars, are more common than to the maxilla and greater tendency toward metastatic colonization has been reported in the mandible compared to other craniofacial bones (Hirshberg et al. 2014; Lim et al. 2006; Lee and Lee 2017; Hirshberg, Leibovich, and Buchner 2008, 1994; Kaplan et al. 2019; Thiele et al. 2011; D'Silva et al. 2006; Ho et al. 2022; Hirshberg, Leibovich, and Buchner 1993). One reason could be remnants of hematopoietic bone marrow in the posterior of the mandible. In patients with focal bone marrow osteoporosis, especially the elderly, active hematopoietic areas are favorable sites for metastases, even though the jaw has little active bone marrow (Hirshberg et al. 2014; Kumar and Manjunatha 2013). In addition, the sinusoidal and perplexed morphology of the vascular spaces in the hematopoietic tissue of the posterior mandible facilitates the establishment of metastatic cells (Batson 1940; Hirshberg et al. 2014; Kaplan et al. 2019; Jham et al. 2011; Ho et al. 2022). Moreover, the anterior of the mandible and the soft tissue of the oral cavity are also involved in advanced stages of the disease (Schwartz, Mignogna, and Baredes 1988).

According to most studies, the gingiva is the most common site of involvement among oral soft tissue metastases (Hirshberg et al. 2014; Seoane et al. 2009; Tezal et al. 2009; Allon et al. 2014). In the study by Seoane et al. on 39 patients in Spain, gingival metastases constituted 67% of all soft tissue metastases in the oral cavity. In the case of metastases to the jaw, the mandible is most frequently affected, whereas in the case of gingival metastases, the maxilla is more frequently affected (Seoane et al. 2009). There is a significant association between gingival metastasis and the presence of teeth (Allon et al. 2014), confirming the role of inflammation in attracting metastatic cells to chronically inflamed gingiva (Lim et al. 2006; Ho et al. 2022; Polligkeit et al. 2006). Remote inflammatory tissues to the primary tumor act as an appropriate pre‐metastatic site (Spano and Zollo 2012; Borsig et al. 2014; Elinav et al. 2013). Chronic inflammation is associated with many stages of invasion and metastasis. In addition, the capillary network of chronically inflamed gums plays an important role in taking up malignant cells (Nagy et al. 1989). Chronically inflamed gingiva with cytokines such as TNF alpha and Interleukin‐1 acts as a favorable site for colonization and metastatic growth (Hirshberg et al. 2014; Seoane et al. 2009; Ho et al. 2022; Tezal et al. 2009; Allon et al. 2014; Aggarwal et al. 2006; Mantovani 2005).

An important characteristic of oral soft tissue metastatic lesions is that they can mimic the appearance of benign neoplastic, inflammatory, or reactive lesions, including PG, peripheral giant cell granuloma (PGCG), peripheral ossifying fibroma, and hemangiomas or vascular malformations (Lim et al. 2006; Nawale et al. 2016; Bodner et al. 2006; Barnes 2009; Hirshberg, Leibovich, and Buchner 1993; Rim et al. 2003). In our study, the gingiva was the most affected area in soft tissue metastases of the oral cavity with jaw involvement in two cases. In addition, there was one case of metastasis to the tongue, although muscles are usually resistant to metastases. As a vascular tissue, the tongue facilitates the uptake of tumor cells and, according to many studies, is one of the most common sites of metastases in oral soft tissues (Hirshberg et al. 2008; Kim, Perry, and Levy 1979; Zegarelli et al. 1973).

It is noteworthy that metastases in the soft tissue of the oral cavity are easier to detect, whereas metastatic lesions in the jaw, particularly in patients with advanced disease, can be diagnosed late, which can result in a poor prognosis until the lesion is fully identified (Hirshberg et al. 2008).

The clinical manifestation of metastatic lesions in the oral cavity varies depending on the localization. Most patients complain of swelling, pain, and paresthesia in the jaw, which develops in a relatively short time, as well as wounds, bleeding, and pathological fractures (Hirshberg et al. 2008; Sánchez‐Jiménez et al. 2005). In addition, condyle lesions are associated with trismus (Hirshberg et al. 2014; Kumar and Manjunatha 2013). Sometimes, there is also tooth dislocation (Reyes Court, Encina, and Levy 2007). In most cases, the metastatic lesions in the gingiva appear as an exophytic mass (96%) that is diagnosed clinically as a reactive gingival lesion (Hirshberg et al. 2014; Allon et al. 2014). Metastatic lesions appear in other oral soft tissue in the form of a submucosal mass, particularly the tongue, and in some cases, appear as ulcers (Hirshberg et al. 2008).

In some cases, metastases are discovered where a tooth was recently extracted. Hirshberg et al. (2008) reported in their review article that in 56 cases, tooth were extracted before metastatic lesions were discovered, resulting in symptoms such as pain, swelling, and loosening of the teeth. Tooth extraction can act as a driving factor in metastasis and cause the spread of metastatic lesions. One reason may be the creation of an environment rich in growth factors (Kumar and Manjunatha 2013; Hirshberg et al. 2008; Clausen and Poulsen 1963; Hirshberg, Leibovich, and Buchner 1994; Hirshberg and Buchner 1995; Tamiolakis et al. 2007; Irani 2016).

In our study, the most common manifestations were pain and swelling, and in some cases, the appearance of ulcers. The intense pain is difficult to control, due to different chemical factors produced by cancer cells, cells derived from the bone marrow, and inflammatory cells. Other symptoms included spontaneous or stimulated bleeding, loose teeth, paresthesia and sensory motor disorders, trismus, restricted mouth opening, and systemic symptoms such as severe weight loss, lethargy, weakness, anorexia, and anemia. An important aspect of paresthesia is that it can be the first symptom of metastasis in 30% of patients. Numb chin syndrome occurs with metastatic tumors in the mandible (Friedrich 2010).

Mental nerve paresthesia (numb chin syndrome) should raise the suspicion of malignancy on clinical examination. It occurs late in the course of the disease because of mental nerve pressure, tumor invasion, or base skull involvement (Lossos and Siegal 1992). Schwartz et al. reported alveolar nerve anesthesia for 50% of mandibular metastasis (Schwartz, Mignogna, and Baredes 1988), whereas Clausen and Poulsen (1963) reported this for only 25% of cases (Clausen and Poulsen 1963). In the study by Murillo et al. (2013) on metastatic tumors to the oral cavity, all patients who had metastatic lesions exclusively in bone or soft tissue involving bone presented symptoms of sensory changes, which, in many cases, resulted in patient referral (Murillo et al. 2013). In the present study, neurological symptoms such as paresthesia occurred in 30% of the patients.

According to a recent review by de Carvalho Kimura et al. (2022), the most common sites of primary cancers metastasizing to the oral cavity are the lung, kidney, liver, thyroid, and prostate (de Carvalho Kimura et al. 2022). Ho et al. (2022) reported the lung to be the most common primary tumor location in both sexes, followed by the colon, kidney, prostate, and pancreas in men and the colon, breast, and kidney in women (Ho et al. 2022). The study carried out by Lim et al. (2006) on soft tissue metastases of the oral soft tissue and jaws reported the liver as the most common site of primary malignancy in the Korean population, followed by the lung, thyroid, female genitalia, and colorectum (Lim et al. 2006). In the studies on populations from western countries, in comparison to eastern countries, mostly the breast has been the most common primary tumor in women. Lim et al. and Nishimura et al. reported, respectively, thyroid and choriocarcinoma as the most common primary tumors in women (Lim et al. 2006; Lee and Lee 2017; Nishimura et al. 1982). The prevalence of primary tumor can vary according to ethnic origin. In a 2015 study on head and neck metastases in an Iranian population (Sadri et al. 2015), only four cases of oral metastases were found, which cannot serve as a basis for comparison.

In our study of oral and maxillofacial metastases in an Iranian population, the kidney was the most common primary tumor site in all patients, followed by the lung, colon, breast, and esophagus, with almost the same prevalence (two cases each, 11.1%). In addition, the most common site of the primary tumor in men and women was the kidney and breast, respectively. Other primary tumors were in the adrenal gland, thymus, thyroid, stomach, leg, and prostate (one case each, 5.5%). Interestingly, all kidney‐originating metastases occurred in men. Kidney cancer has a high prevalence as the origin of oral cavity metastases. It has also been reported that renal cell carcinoma tends to spread aggressively in the early stages (van der Waal, Buter, and van der Waal 2003).

Certain tumors tend to metastasize to certain sites. Studies have shown that a number of primary tumors such as breast, prostate, thyroid gland, and kidneys tend to metastasize to bone (Ho et al. 2022; Wells et al. 2013; Coleman 2006). Hirshberg et al. (2008) reported that 11% of jaw metastases were from the prostate compared to 1.5% of oral soft tissue metastases and that 40% of jaw metastases were from the breast compared to 25% of oral soft tissue metastases (Hirshberg et al. 2008). According to Ho et al., in addition to primary breast and prostate tumors, a strong tendency to metastasize in cancers of the adrenal glands and female genitalia was also reported. Moreover, lung tumors tended to metastasize to the oral soft tissues and were the most common in both sexes (Ho et al. 2022). Physicians and dentists are therefore advised to pay particular attention to more such common areas during regular clinical examinations (Jham et al. 2011).

Ho et al. proposed that tumors metastasizing to the oral cavity are generally the most common malignancies in humans, such as malignancies of the lungs, which, according to their retrospective cohort study with 40 cases, occurred most frequently in both sexes. In addition, on examining 1084 cases, primary lung and breast tumors were the most common malignancies in men and women, respectively (lung cancer is more common in men than women, and breast cancer is rare in men). However, there are also some exceptions. Although malignancies in the kidney and renal pelvis accounted for only 4% of all cancers in America, with an estimated 76,084 new cases in 2021 (Ho et al. 2022), renal metastases ranked second among the most common metastatic lesions in the oral cavity. Kidney metastases were observed more than prostate and colorectum metastases, whereas kidney malignancy was less common in cancers. According to Ho et al., such discrepancies may be due to differences in the biological behavior and aggressiveness of the primary tumor, as well as the tendency for each cancer to spread to specific parts of the body (Hirshberg et al. 2008, 2014; Lim et al. 2006; Ho et al. 2022).

Furthermore, Ho et al. reported that bone and smooth muscle sarcomas (7%) and pancreatic carcinoma (2%) were the rarest cases of oral cavity metastases, probably due to the death of patients before metastasis to the oral cavity because of the poor prognosis of the primary tumor (Ho et al. 2022). Interestingly, we also found a case of a 13‐year‐old patient with osteosarcoma.

Oral metastases are very unlikely in people younger than 40 years of age and are usually associated with neuroendocrine neoplasms (Shimono et al. 2021; Owosho et al. 2016; Aniceto et al. 1990). In addition to the previously mentioned case of a 13‐year‐old patient, our study also found three other patients younger than 40 years of age: one with thymus carcinoma (34 years old), one with invasive ductal carcinoma in the breast (29 years old), and one with neuroblastoma (6 years old).

As in recent studies, the vast majority of cases were carcinomas and particularly adenocarcinomas (de Carvalho Kimura et al. 2022), probably because sarcomas are rare compared to epithelial malignancies (Kaplan et al. 2019). According to studies, the diagnosis of malignant metastasis led to the detection of the primary tumor in 25%–33% of cases of metastasis to the oral and maxillofacial region (Hirshberg, Leibovich, and Buchner 1993, 1994; Maschino et al. 2013; Polligkeit et al. 2006; Adelson et al. 2005). In our study, the primary tumor was occult in six cases and was discovered after diagnosis of the metastatic lesion of the oral cavity.

The survival rate in patients with metastases to the oral cavity is low. According to many studies, oral metastasis indicates advanced disease with extensive metastases (Kumar and Manjunatha 2013; Hirshberg, Leibovich, and Buchner 1994; Meyer and Shklar 1965). Shimono et al. (2021) reported a 1‐year survival rate after diagnosis of oral metastatic tumors in Japan of 30% (Shimono et al. 2021). The mean survival has been reported in studies to be 6–8 months (van der Waal, Buter, and van der Waal 2003; Murillo et al. 2013; Sánchez‐Jiménez et al. 2005). Follow‐up information was not fully available in this study. However, in those who died, death occurred within a year or less of diagnosis of the primary tumor.

Treatment depends on several factors, including the location of the lesion, the site of the primary tumor, and the degree of metastatic spread (Aniceto et al. 1990). Treatment includes surgical resection, radiotherapy, chemotherapy, and palliative treatments (Irani 2017). Most patients with metastatic tumor in the oral cavity also develop metastases elsewhere, often leaving no other choice than palliative and conservative treatment to maintain the patient's quality of life and functions (van der Waal, Buter, and van der Waal 2003). Despite advances in cancer treatment, early diagnosis has a significant impact on improving prognosis. In 20%–35% of cases, the oral and maxillofacial metastatic lesion is the only focus of distant metastasis. In such cases, rapid and regular systemic examinations should prevent the disease from progressing to higher stages, including multifocal metastases, and improve the prognosis (Lee and Lee 2017; de Carvalho Kimura et al. 2022). Therefore, dentists play an important role in the diagnosis of patients with metastatic lesions in the oral cavity. The diagnosis of metastasis should be considered among other diagnosis, particularly in patients with previous malignancies (de Carvalho Kimura et al. 2022).

## 5 Conclusion

Most of the results of this study were demographically consistent with those of other studies. Metastases to the oral cavity and jaw were more common in men. The lesions appeared in the fourth to sixth decade of life and were more common in the jaw, especially the mandible, than in the oral soft tissues. According to other studies, oral and maxillofacial metastases are rare and have no specific symptoms. Due to the different clinical appearances of such metastases, they can easily be confused with benign and inflammatory lesions. The physicians should consider the possibility of metastasis of the lesion, especially if the patient has a history of malignancy. Early diagnosis is therefore vital and helps improve the prognosis.

## Author Contributions

Conceptualization: Atessa Pakfetrat. Acquisition of data: Sajede Asalian and Armaghan Salehi. Analysis and interpretation of data: Sajede Asalian, Atessa Pakfetrat, Nasrollah Saghravanian, Zohreh Dalirsani, and Kazem Anvari. Statistical analysis: Mahboobeh Taherizadeh. Drafting the manuscript: Sajede Asalian. Critical revision for important intellectual content: Atessa Pakfetrat, Zohreh Dalirsani, Nasrollah Saghravanian, and Kazem Anvari. Study supervision: Atessa Pakfetrat, Zohreh Dalirsani, and Sajede Asalian. All authors critically revised the manuscript and approved the final revision.

## Ethics Statement

The study protocol was approved by the Research and Ethics Committee of Mashhad University of Medical Sciences (IR.MUMS.DENTISTRY.REC.1400.027).

## Consent

In this study, the identity of none of the patients has been revealed and codes have been used to introduce the patients; also, all the personal information of the patients remains confidential with the researcher. In addition, informed consent was obtained from all patients of this study before obtaining the medical records.

## Conflicts of Interest

The authors declare no conflicts of interest.

## Supporting information

Supporting information.

## Data Availability

The data that support the findings of this study are available on request from the corresponding author. The data are not publicly available due to privacy or ethical restrictions.

## Endnote

1 C79.8 (secondary malignant neoplasm of other specified site) and C79.5 (secondary malignant neoplasm of bone and bone marrow) were the codes used to extract patient records according to the ICD 10 book, 2016 edition. This system has been used as a reference for coding in the medical records department of Iranian hospitals.
