# Supplementary material for: Tumor Metastasis to the Oral Soft Tissues and Jaw Bones: A Retrospective Study and Review of the Literature
Source: Clin Exp Dent Res. 2024 Oct 17;10(6):e70011. doi: 10.1002/cre2.70011 (PMC11486913; doi:10.1002/cre2.70011)
Supplement: Supplementary file 1 — Supporting information. [file CRE2-10-e70011-s001.docx]

|  | | | Metastatic tumor | | | | | | Primary tumor | | |  | | |
| --- | --- | --- | --- | --- | --- | --- | --- | --- | --- | --- | --- | --- | --- | --- |
| Follow up  Results ^^[[1]](#footnote-1)^^ | Metastasis to other organs | Time interval^^[[2]](#footnote-2)^^ | Treatment^^[[3]](#footnote-3)^^ | Pathologic diagnosis | Radiologic features | Symptoms | Signs | Location | Treatment^^[[4]](#footnote-4)^^ | Pathologic diagnosis | Primary  site | Age | Sex |  |
| 1 year/ disease  Free | Not having | 22 months | Surgery/ Chemo therapy | Squamous Cell Carcinoma | ------ | Without pain | Mass / firm consistency | Tongue | Chemo therapy/  radio therapy | Squamous Cell Carcinoma | Eophagous | 54 | Fe | 1 |
| 1 year/ died | Spinal bones/اskeletal bones/ abdominal and cervical lymph nodes | 1 month | Palliative treatment | Adeno-  carcinoma | -------- | ----- | ------ | Maxillofacial | Palliative treatment | Adeno-  carcinoma | Prostate | 62 | M | 2 |
| ------- | Not having | 2 years | Surgery | Pheochromo  cytoma | Osteogenic Radiolucency/ sunray periosteal reaction | Without pain/ lip Insensibility/ systemic signs^^[[5]](#footnote-5)^^ | Expansion | Mandible | Surgery | Pheochromo  cytoma | Adrenal gland | 45 | Fe | 3 |
| --- | -- | ---- | Surgery | Metastasis of Ductal Carcinoma of Breast | -------- | -- | -- | Mandible and gingiva | ---- | Ductal Carcinoma | Breast | 48 | Fe | 4 |
| 6 months/ died | Liver | 3 years | Chemo therapy | Adenocarcinoma | Healthy | Pain^^[[6]](#footnote-6)^^/ bleeding^^[[7]](#footnote-7)^^ | Mass/rubbery consistency/granular surface/telangiectatic | Upper jaw gingiva | Surgery | Adenocarcinoma | Colon | 60 | Fe | 5 |
| ---- | ----- | ---- | ---- | Metastatic Poorly Differentiated Adenocarcinoma of Lung | ----- | Insensibility and paresthesia/  Tooth mobility | ---- | Lower lip | **** | ------ | Lung | 48 | M | 6 |
| ---- | --- | 1 year | ---- | Small Round Cell Tumor compatible with: 1-Ewing sarcoma 2- Small cell Osteosarcoma | -------- | -- | mass | Mandible | Surgery | Osteosarcoma | leg | 13 | M | 7 |
| 9 months  / died | Lungs/ brain^^[[8]](#footnote-8)^^ | ***** | Surgery/ Chemo therapy/ radio therapy | Renal Cell Carcinoma | Radiolucency | pain | Mass without wound/ firm consistency/ mauve color | maxilla | Surgery ^^[[9]](#footnote-9)^^ | Renal Cell Carcinoma | Kidney | 75 | M | 8 |
| Died | -------- | ------ | Chemo therapy | Thymic carcinoma | ----- | --- | ----- | Maxilla | ---- | Thymic Carcinoma | Thymus | 34 | M | 9 |
| ---- | ---------- | 13 years | Surgery | Follicular Carcinoma of Thyroid | ---- | --- | ---- | Mandible | Surgery | Follicular Carcinoma | Thyroid | 59 | Fe | 10 |
| 1 year / died | --- | 7 years | --- | Squamous Cell Carcinoma g II | -------- | --- | Wound | Mandible and gingiva | Surgery/ Chemo therapy/  radio therapy | Squamous Cell Carcinoma | Esophagus | 52 | M | 11 |
| ------- | ----- | 2 years | Surgery | Adenocarcinoma | ---- | Malfunction of facial nerve | Mass | Buccal vestibule | Chemo therapy | Adenocarcinoma | Stomach | 83 | M | 12 |
| ---- | Spinal bones/hips/ribs | **** | Chemo therapy | Metastatic Carcinoma of breast | Ill-defined radiolucency | Pain/  Paresthesia/ neurologic malfunction/^^[[10]](#footnote-10)^^ systemic signs  ^^[[11]](#footnote-11)^^ | Swallow/ wound on surface^^[[12]](#footnote-12)^^ | Mandible | ***** | Invasive Ductal Carcinoma | Breast | 29 | Fe | 13 |
| ----- | ---- | 1 year | Radio therapy | Metastatic  Adenocarcinoma | Ill-defined radiolucency | Pain/ tooth mobility/tend to bleeding | Mass /firm consistency | maxilla | Surgery/ Chemo therapy/  radio therapy | Adenocarcinoma | Colon | 54 | M | 14 |
| Remission | Not having | 2 years | Surgery Chemo therapy | ^^[[13]](#footnote-13)^^ neuroblastoma | Radiolucency ^^[[14]](#footnote-14)^^ | Tooth mobility | Swallow/expansion/ rubbery, soft, bony consistency in different areas / mauve color | Mandible and sub mandible | Surgery/ Chemo therapy | Neuroblastoma | Kidney | 6 | M | 15 |
| 1 month/ died | Liver/ scapula/ para-aorta | 8 months | Chemo therapy | Metastasis of Malignant Kidney Tumor | Radiolucency | Without pain/ limitation of mouth opening | Swallow/firm consistency | Mandible and parotid | Surgery | Renal Cell Carcinoma ^^[[15]](#footnote-15)^^ | Kidneys | 54 | M | 16 |
| Still receiving treatments | اBones /liver^^[[16]](#footnote-16)^^ | ****** | Radio therapy/  Chemo therapy | Metastatic Adenocarcinoma of Lung | Ill-defined radiolucency | ---- | Swallow | Mandible | ***** | Non-small Cell Lung Cancer ^^[[17]](#footnote-17)^^ | Lungs | 59 | Fe | 17 |
| ----- | Not having | ***** | Surgery/ other treatments^^[[18]](#footnote-18)^^ | Metastatic Renal Clear Cell Carcinoma | Ill-defined radiolucency | Tooth mobility/ trismus | Mass/soft consistency | Mandible | Surgery^^[[19]](#footnote-19)^^ | Clear cell Carcinoma (conventional Renal Cell Carcinoma) | Kidneys | 57 | M | 18 |

Table 1. Detailed presentation of cases. Demographic and some clinical properties of metastatic cases.

The rows that are colored in the table are those cases with undiscovered primary cancer, that the diagnosis of oral metastasis led to discovery of primary cancer. Therefore, factors such as initial treatment and time interval are not defined for them and the relevant parts in the table are filled with this mark (*).

* Case number 1 has been reported before. ( Aledavood, Akbari Oryani, and Pishevar Feizabad 2022)

** Case number 3 has shown in figure 1. (Rahpeyma and Khajehahmadi 2020)

*** Case number 5 has shown in figure 2. (Dalirsani, Mohtasham, and Samiee 2020)

**** Case number 8 has shown in figure 3. (Saghravanian, Rajaei, and Bashardoust 2010)

***** Case number 13 has shown in figure 4

****** Case number 14 has shown in figure 5 *******

1. Time period that patient had been following up after diagnosis of metastatic lesion and the final destination followed by such as death, remedy... [↑](#footnote-ref-1)
2. Time period between diagnosis of primary and oral metastatic tumor. [↑](#footnote-ref-2)
3. The treatments received after diagnosis of oral metastatic tumor. [↑](#footnote-ref-3)
4. The treatments received after diagnosis of primary tumor. [↑](#footnote-ref-4)
5. Anemia / severe sweeting. [↑](#footnote-ref-5)
6. Tenderness. [↑](#footnote-ref-6)
7. Bleeding during brushing teeth. [↑](#footnote-ref-7)
8. Based on the findings of CT-scan. [↑](#footnote-ref-8)
9. Nephrectomy was performed after diagnosis of jaw metastasis. [↑](#footnote-ref-9)
10. Inability to close the left eyelid. [↑](#footnote-ref-10)
11. Weight loss / weakness / pallor / anorexia / feeling pain in back, chest and legs. [↑](#footnote-ref-11)
12. As a result of opposite third molar trauma. [↑](#footnote-ref-12)
13. . Two biopsies had been taken for this lesion with results of calcifying odontogenic cyst and round cell tumor which non of them were true and because the whole sample had been removed during surgery, another try for test was impossible. According to complete examinations, metastasis of neuroblastoma.

    considered as the diagnosis of jaw lesion. [↑](#footnote-ref-13)
14. Disappearing of inferior alveolar canal walls/ resorption of bone crypt in upper left dental molar buds area. [↑](#footnote-ref-14)
15. Based on the investigations carried out, the sickness period of this patient is as follows: the patient underwent right kidney removal surgery, according to pathology diagnosis of nonfunctional kidney. Then the swelling of mandibular and parotid area appeared and dental abscess as the primary diagnosis ruled out and the presence of malignancy was suggested. After further investigations and discovering multiple distant metastatic lesions, the diagnosis of renal cell carcinoma strongly was suggested for this patient. [↑](#footnote-ref-15)
16. Based on bone scan report and CT scan [↑](#footnote-ref-16)
17. The findings of spiral CT scan strongly suggested Non-Small Cell Lung Cancer and the right lung as the primary origin. [↑](#footnote-ref-17)
18. Unfortunately, its details weren’t available [↑](#footnote-ref-18)
19. After the primary tumor is discovered and followed by diagnosis of jaw metastasis. [↑](#footnote-ref-19)
